# Improved Charge Carrier Dynamics by Unconventional Doping Strategy for BiVO_4_ Photoanode

**DOI:** 10.1002/smsc.202500051

**Published:** 2025-05-19

**Authors:** Jiseok Kwon, Heechae Choi, Seunggun Choi, Jooheon Sun, Hyuksu Han, Ungyu Paik, Junghyun Choi, Taeseup Song

**Affiliations:** ^1^ Department of Energy Engineering Hanyang University 222 Wangsimni‐ro Seongdong‐gu, Seoul 04763 Republic of Korea; ^2^ Department of Chemistry Xi'an Jiaotong‐Liverpool University Suzhou 215123 P. R. China; ^3^ Department of Energy Science Sungkyunkwan University 2066 Seobu‐ro Jangan‐gu, Suwon‐si, Gyeonggi‐do 16419 Republic of Korea; ^4^ Division of Materials Science and Engineering Hanyang University 222 Wangsimni‐ro, Seongdong‐gu Seoul 04763 Republic of Korea; ^5^ School of Chemical, Biological and Battery Engineering Gachon University Seongnam‐si, Gyeonggi‐do 13120 Republic of Korea; ^6^ Department of Battery Engineering Hanyang University 222 Wangsimni‐ro Seongdong‐gu, Seoul 04763 Republic of Korea

**Keywords:** BiVO_4_, carrier lifetime, diffusion length, doping, space charge layer, water oxidation

## Abstract

Bismuth vanadate (BiVO_4_) is one of the promising photoanodes for solar fuel production, but it faces the challenge of poor charge separation due to its sluggish charge transport and short diffusion length. The ability to regulate charge separation is pivotal for obtaining high efficiency of BiVO_4_. Herein, an unconventional acceptor doping strategy is proposed for the first time, demonstrating its effectiveness in enhancing charge carrier dynamics. Introducing the Al^3+^ ions into BiVO_4_ induced a decrease in carrier concentration but an increase in the diffusion length and carrier lifetime due to the reduced chance of encountering an electron‐hole pair. Furthermore, decreasing carrier concentration leads to a widened space charge layer, enabling facile charge transport and separation. The optimized 0.5 at% Al‐doped BiVO_4_ (Al:BVO_0.5) exhibited ≈3.5 and 2.6 order of magnitude increase in diffusion length and in carrier lifetime, respectively, compared to pristine BiVO_4_, achieving a photocurrent density of 3.02 mA cm^−2^ at 1.23 *V*_RHE_ (V versus reversible hydrogen electrode) under AM 1.5 G illumination. This research provides a new understanding of semiconductor physics and design principles for more efficient photoanodes.

## Graphical abstract

An unconventional acceptor doping strategy using Al ions in Bismuth vanadate (BiVO_4_) improves the charge carrier dynamics of BiVO_4_. By reducing carrier concentration and widening the space charge layer, Al doping enhances diffusion length and carrier lifetime. This leads to more efficient charge separation in both bulk and surface, offering a promising strategy for advanced photoanode design.

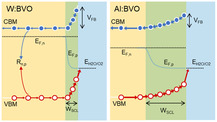

## 1 Introduction

Photoelectrochemical (PEC) water splitting has gained significant attention as a promising strategy for utilizing solar energy to produce hydrogen and oxygen.^[^ ^1^, ^2^, ^3^ ^]^ Among the various photoabsorbers studied, bismuth vanadate (BiVO_4_), particularly in its monoclinic structure, stands out as one of the most promising n‐type semiconductors.^[^ ^4^, ^5^, ^6^ ^]^ Monoclinic BiVO_4_ features a 2.4 eV bandgap, enabling visible light absorption, and has favorable conduction and valence band positions for the water splitting reaction.^[^ ^7^, ^8^ ^]^ Despite these advantageous properties, BiVO_4_ photoanodes have suffered limitations such as poor electrical conductivity, short carrier diffusion length, and slow water oxidation kinetics.^[^ ^9^, ^10^ ^]^ These lead to poor charge separation within the bulk material and at the interface during PEC water splitting, significantly hindering the PEC performance of BiVO_4_ photoanodes.^[^ ^11^ ^]^

To overcome the intrinsic limitations of BiVO_4_, extrinsic doping of donor ions, such as Mo^6+^ and W^6+^, has been applied to increase carrier concentration, thereby improving the electrical conductivity and PEC performance.^[^ ^12^, ^13^, ^14^ ^]^ Introducing donor ions in BiVO_4_ reduces the surface charge transfer resistance, facilitating water oxidation.^[^ ^15^ ^]^ However, with higher carrier concentration, impurity scattering becomes more significant, resulting in a reduction in carrier mobility.^[^ ^16^, ^17^ ^]^ Higher carrier concentrations can also lead to increased recombination events, which shorten the carrier lifetime and the diffusion length. Furthermore, donor ions act as trap sites, adversely affecting charge carrier transport.^[^ ^18^ ^]^ For example, Abdi et al. reported that the hole diffusion length of *W*‐doped BiVO_4_ was 23 nm, which is decreased compared to that of BiVO_4_ (70 nm), suggesting W^6+^ doping strongly decreases the carrier mobility by introducing intermediate‐depth donor defects as carrier traps.^[^ ^19^, ^20^ ^]^

The separation of photogenerated electron‐hole pairs (EHP) is significantly influenced by the width of the space charge layer (*W*_SCL_), which is formed as a result of band bending.^[^ ^21^ ^]^ The band bending creates a localized electric field, which is essential for driving the separation of EHP and preventing recombination.^[^ ^22^ ^]^ As a result, increasing *W*_SCL_ facilitates the separation of EHP. However, incorporating donor ions such as W^6+^ and Mo^6+^ in BiVO_4_ decreases the *W*_SCL_, limiting it to a few nanometers.^[^ ^14^, ^23^ ^]^ EHP in this thin SCL has a higher chance of recombining before contributing to the desired PEC reaction. Furthermore, since the material absorbs light at a greater distance than the SCL can manage, recombination of EHP generated deeper within the material is unavoidable, so a thin SCL limits the effective utilization of incident light. Therefore, this limitation necessitates strategies to expand SCL to improve charge transport and enhance light absorption closer to the surface.

Based on these, lowering the carrier concentration in BiVO_4_ could be an effective strategy for improving charge carrier dynamics and PEC performance.^[^ ^24^, ^25^ ^]^ Lower carrier concentration reduces the probability of recombination as the charge carriers are less likely to encounter each other within a given period. In addition, a reduced carrier concentration can lead to an increase in *W*_SCL_, enabling more charge carriers to reach the surface where they can participate in PEC reactions. However, the lower carrier concentration could reduce the electrical conductivity, making it harder for charge carriers to reach the reaction sites. The relationship between carrier concentration and charge carrier dynamics is a delicate balance. Optimizing one aspect requires trade‐offs in another. Therefore, a careful tuning strategy of carrier concentration to balance these competing factors is necessary to achieve maximum performance of BiVO_4_.

Herein, we present the unconventional acceptor ions doping strategy to improve the charge carrier dynamics and PEC performance of BiVO_4_. This study aims to comprehensively understand how carrier concentration modulation can be leveraged to increase the diffusion length and carrier lifetime. Introducing Al acceptor ions into BiVO_4_, which substitutes V sites, increases the diffusion length and carrier lifetime even with a reduced carrier concentration. The optimal 0.5 at% Al‐doped BiVO_4_ (Al:BVO_0.5) exhibited ≈3.5 and 2.6 order of magnitude increase in diffusion length and carrier lifetime, respectively, compared to BiVO_4_. Furthermore, the reduced carrier concentration resulted in a widened SCL, enabling the facile charge transport. Improved charge carrier dynamics by Al acceptor ion doping boosts the charge separation both in bulk and on the surface, leading to superior PEC performance. The Al:BVO_0.5 achieves a photocurrent density of 3.02 mA cm^−2^ at 1.23 *V*_RHE_ (V versus reversible hydrogen electrode), which is superior to W‐doped BiVO_4_. Our findings not only offer insights into fundamental semiconductor physics but also propose a viable strategy for optimizing semiconductors for highly efficient photoanodes.

## 2 Result and Discussion

### 2.1 Materials Characterization

**Figure 1:**
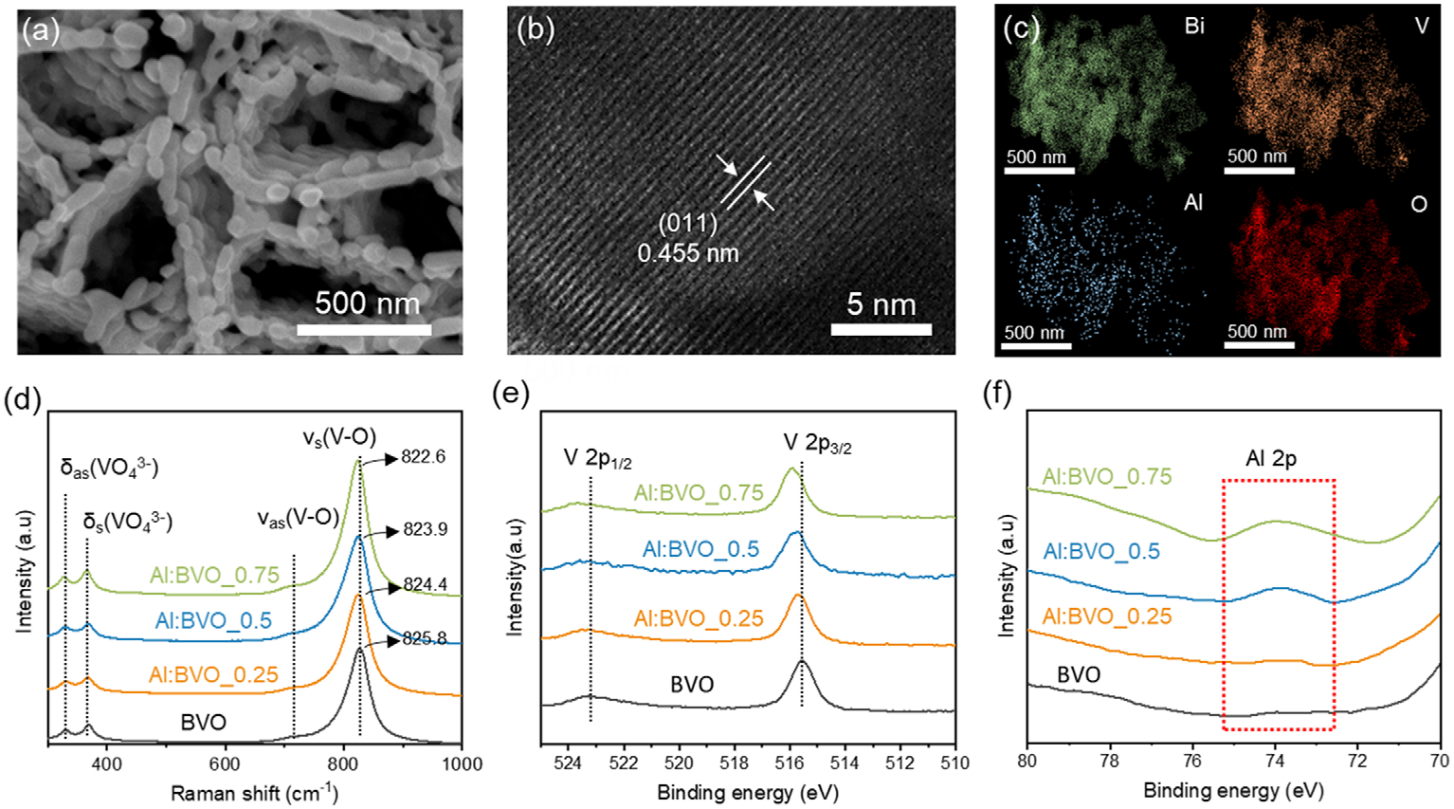
a) SEM image, b) High resolution TEM image, and c) EDS elemental mapping of Al:BVO_0.5. d) Raman spectra of BVO and Al:BVOs. e) V 2p, f) Al 2p XPS spectra of BVO and Al:BVOs.

The pristine BiVO_4_ (BVO) and Al‐doped BiVO_4_ (Al:BVOs) with doping concentrations of 0.25, 0.5, and 0.75 at% were fabricated via electrodeposition and conversion method. The top‐view scanning electron microscopy (SEM) image of Al:BVO_0.5 in **Figure** **1**a shows a nonporous structure with flake frames. It is confirmed that the morphological change with Al doping concentration is negligible (Figure S1, Supporting Information). The cross‐sectional view SEM image, in Figure S2, Supporting Information, shows BiVO_4_ nanostructure with a thickness of 700 nm. The crystal structure of Al:BVOs was investigated by X‐ray diffraction (XRD) measurements. Figure S3, Supporting Information shows that XRD patterns of BVO and Al:BVOs correspond to monoclinic scheelite structure (JCPDS card 14‐0688) regardless of doping concentration. Magnified XRD view shows that as the doping concentration increases, the peak, corresponding to (121) plane, observed at 28.90, 28.96, 28.97, and 28.98 θ for BVO, Al:BVO_0.25, Al:BVO_0.5, and Al:BVO_0.75, respectively. This shift to the higher value of 2θ indicates lattice contraction due to the smaller ionic radius of Al^3+^ (0.0535 nm) compared to V^5+^ (0.059 nm).^[^ ^26^, ^27^ ^]^ High‐Resolution Transmission Electron Microscopy (HR‐TEM) image of Al:BVO_0.5 in Figure 1b shows lattice fringes with the spacing of 0.455 nm, corresponding to (0 1 1) plane, which is smaller than that of BVO (0.462 nm) in Figure S4, Supporting Information. These HR‐TEM results coincide with the XRD results. TEM energy‐dispersive spectroscopy (EDS) mapping images show a homogeneous distribution of Bi, V, O, and Al elements in Al:BVO_0.5, demonstrating the presence of Al (Figure 1c).

Raman spectroscopy was employed to investigate the bonding feature of Al:BVO. As shown in Figure 1d, the band positions around 825.8 and 716.2 cm^−1^ of the BVO were assigned to the symmetric stretching *ν*_s_ (V—O) mode and asymmetric stretching *ν*_as_ (V—O) modes. Symmetric *δ*_s_ (VO_4_^−3^) and asymmetric *δ*_as_ (VO_4_^−3^) bending vibrations were detected at 363.4 and 326.4 cm^−1^, respectively.^[^ ^28^ ^]^ As the doping concentration increases, the *ν*_s_ (V—O) shifts to a lower wavenumber, indicating the weakness and elongation of V—O bond of Al:BVOs. When Al is introduced in BiVO_4_, since ionic radii of Al^3+^ is smaller than V^5+^, it introduces lattice distortion and weaker Al—O bonds compared to V—O bonds. This induces a redshift in the Raman spectra due to weaker Al—O bond strength. Furthermore, oxygen vacancies reduce the number of V—O bonds, weakening the bond network and redshift in Raman spectra.^[^ ^29^ ^]^ Electronic structures of Al:BVOs were investigated by X‐ray photoelectron spectroscopy (XPS). Figure 1e and Figure S5a, Supporting Information show a prominent peak shift in V 2p and Bi 4f spectra of Al:BVOs toward higher binding energy compared to the BVO due to different electronegativities of Al^3+^ and V^5+^.^[^ ^30^ ^]^ It indicates the concentration of V^5+^ increases on the surface of BiVO_4_, which creates a built‐in electric field that promotes charge separation and reduces recombination at the BiVO_4_/electrolyte interface.^[^ ^31^ ^]^ In the O 1s spectra (Figure S5b, Supporting Information), strong peaks at 528.5 eV were observed, corresponding with the metal–oxygen binding in BVO lattice.^[^ ^32^ ^]^ As doping concentration increases, the shoulder peak at 530.5 eV increases, which is attributed to the formation of oxygen vacancies (*O*_v_) to maintain charge neutrality. With an increase of V^5+^, these *O*_v_ are ionized *O*_v_ where the electrons have been thermally or photochemically excited to the conduction band, leaving behind positively charged defects. Ionized *O*_v_ in the surface region creates a built‐in electric field, accelerating hole migration to the electrolyte and electron transport to the back contact.^[^ ^33^ ^]^ Figure 1f shows the Al 2p spectra of BVO and Al:BVO. The prominent peak at 73.9 eV appeared as the doping concentration increased in Al:BVO whereas the distinct peak was not formed in the BVO, which further demonstrates the Al incorporation in BVO.

### 2.2 Carrier Concentration and Optical Band Structure

**Figure 2:**
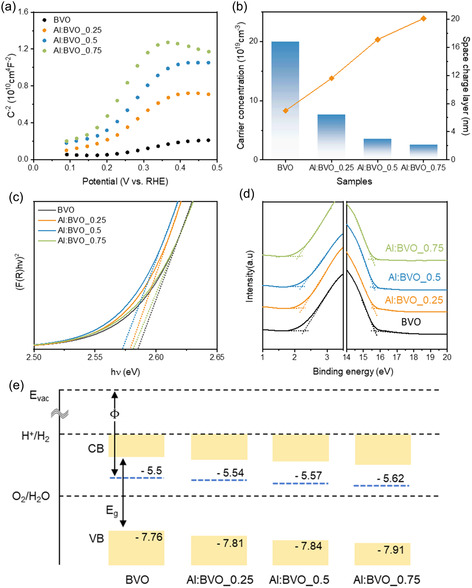
a) Mott–Schottky (M–S) plots of BVO and Al:BVOs measured at 1 kHz. b) Carrier concentration and width of space charge layer derived from M–S plot. c) Tauc plot for determining the optical bandgap of BVO and Al:BVOs. d) UPS spectra of BVO and Al:BVOs. e) Schematic illustration of the band diagram of BVO and Al:BVO.

Mott–Schottky (M–S) analysis was carried out to demonstrate the effect of Al doping on charge carrier concentration (*N*_d_). As shown in **Figure** **2**a, all the samples show a positive M–S plot, which indicates an N‐type characteristic of BVO and Al:BVOs. It was observed that the slope increases with increasing doping concentration, which indicates a decreased carrier concentration. The results derived from M–S plot are summarized in Table S1, Supporting Information, and presented in Figure 2b. As doping concentration increases, *N*_d_ tends to decrease, which is ascribed that intrinsic donors in BVO would be compensated by Al^3+^ acceptors. Decrease in *N*_d_ in Al:BVOs induce the extension of the SCL, where the separation of EHP occurs. The *W*_SCL_ of Al:BVO_0.5 is 17.07 nm, 2.42 times that of BVO (7.04 nm). An increased *W*_SCL_ of Al:BVOs can facilitate charge separation, leading to an increase in PEC performance and efficiency. However, if the layer is too thick due to excessively low *N*_d_, the likelihood of recombination is reduced, while the decreased electrical conductivity due to low *N*_d_ can make it difficult for charge carriers to reach the reaction site. Therefore, optimization of SCL thickness and carrier concentration is required to maximize the PEC performances.

The optical bandgap of Al:BVOs was estimated using UV‐diffuse reflectance spectroscopy (UV‐DRS). The UV‐DRS spectra of BVO and Al:BVOs in Figure S6, Supporting Information, show that all the samples have similar absorption spectra with absorption edges around 480 nm. From the Kubelka–Munk equation, the optical bandgap of BVO and Al:BVOs was determined from the intercept in the Tauc plot in Figure 2c. Similar bandgaps of 2.57 ≈ 2.59 were obtained for all the samples, confirming the negligible effect on band structure by Al doping. In addition, ultraviolet photoelectron spectroscopy (UPS) was employed to analyze the Fermi level (*E*_F_) and valence band maximum (VBM) positions. Figure 2d is the magnified UPS spectra near the valence band and the onset of the secondary emission (*E*_cut–off_). It was observed that the difference between the *E*_F_ and VBM decreased with increasing doping concentration. *E*_cut–off_ value also decreased with a negative shift, indicating the increased work function. The results determined by UPS are presented in Table S2, Supporting Information. The band diagram of Al:BVOs is constructed by work function and the difference between the *E*_*F*_ and VBM, and presented in Figure 2e. As doping concentration increases, the VBM and conduction band minimum (CBM) positions are slightly shifted downward. *E*_F_ is also downshifted to a greater extent than CBM, causing *E*_F_ to move far away from CBM due to decreased *N*_d_.

### 2.3 Charge Carrier Dynamics

**Figure 3:**
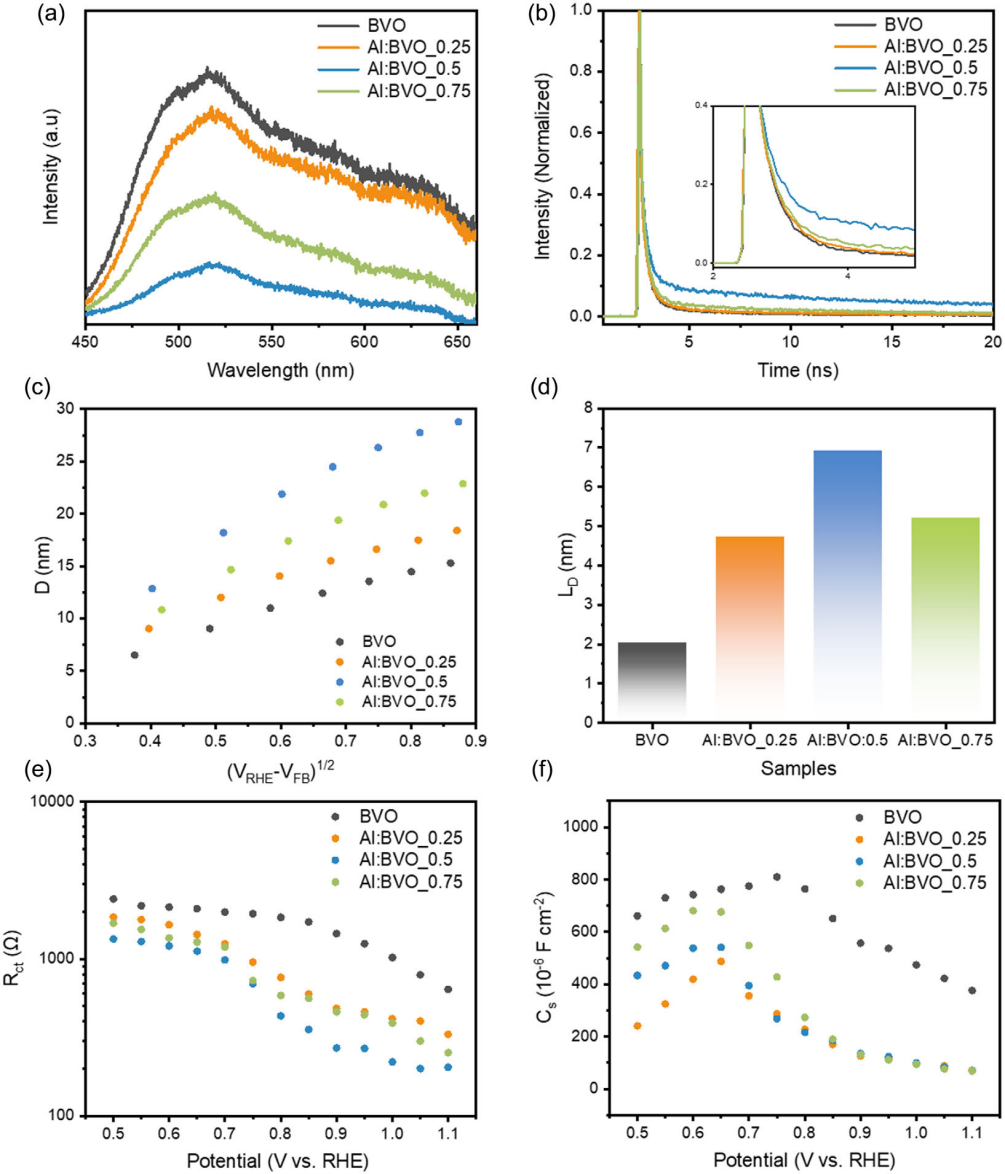
a) PL plots, b) Time‐resolved PL decay of BVO and Al:BVOs. c) Distance D as a function of the square root of the difference between the potential *V*_RHE_ and the flat band potential *V*_FB_. Y‐intercept indicates the diffusion length (*L*_D_). d) The obtained *L*_D_ values of BVO and Al:BVOs. e,f) PEIS measurement. Fitting results of *R*_ct_ and *C*_s_ as a function of applied potential.

To investigate the charge carrier dynamics of Al:BVOs, photoluminescence (PL) was employed (**Figure** **3**a). Generally, a prolonged carrier lifetime ensures a diminished recombination rate, resulting in lower PL intensity. Al:BVOs exhibited a lower PL intensity compared to BVO, which is attributed to suppressed recombination. Al:BVO_0.5 showed the smallest PL intensity among Al:BVOs, indicating the optimal doping ratio for suppressing charge recombination. Time‐resolved PL analysis was also performed. The PL decay profiles in Figure 3b were fitted by a quadruple exponential function. The fitting parameters are summarized in Table S3, Supporting Information. The intensity‐weighted average carrier lifetime (*τ*_aveg_) was 14, 15, 37, and 21 ns for BVO, Al:BVO_0.25, Al:BVO_0.5, and Al:BVO_0.75, respectively, demonstrating that introducing acceptor ions in the BVO lattice increases the carrier lifetime. However, the excess doping concentration leads to a decrease in charge carrier lifetime, which is attributed to the foreign Al species and surface *O*_v_ serving as a recombination site of EHP. Carrier lifetime images of BVO and Al:BVOs are presented in Figure S7, Supporting Information.

The carrier diffusion length (*L*_D_), representing the average distance that EHP can move before they recombine, is another parameter to evaluate the charge carrier dynamics of Al:BVOs.^[^ ^34^ ^]^ The carrier diffusion length was estimated by the transient photocurrent (TPC) Measurement.^[^ ^35^, ^36^ ^]^ Figure 3c shows the distance D versus the square root of the difference between the applied potential and the flat band potential (*V*_FB_). The distance D shows a linear relationship with (*V*_RHE_ − *V*_FB_)^1/2^ and the Y‐intercept represents the *L*_D_ value. Assuming no recombination at SCL occurs, distance D corresponds to the total thickness over which the hole can be utilized to oxidize the electrolyte, expressed as the sum of *W*_SCL_ and *L*_D_. The D value increased with the introduction of acceptor ions, with the Al: BVO_0.5 showing the largest D value among the Al:BVOs. This result is consistent with the increased carrier lifetime of Al:BVOs. The obtained *L*_D_ values in Figure 3d are 2.04, 4.73, 6.93, and 5.21 nm for BVO, Al:BVO_0.25, Al:BVO_0.5, and Al:BVO_0.75, respectively. This increased *L*_D_ allows charge carriers to travel further without recombining, increasing the likelihood that they reach the interface, where they can participate in the PEC reaction.

To further understand the impact of Al doping in BiVO_4_ on the interfacial charge transfer, PEC impedance spectroscopy (PEIS) measurements were performed under applied biases from +0.5 to +1.1 *V*_RHE_. The *R*_ct_ of BVO and Al:BVOs decreases with increasing applied bias, indicating that applied bias can significantly improve charge separation and injection (Figure 3e). The *R*_ct_ of the Al:BVO_0.5 photoanodes shows the lowest value compared to other counterparts, indicating that the improved charge separation and injection can be achieved at a lower bias. As shown in Figure 3f, the surface capacitance (*C*_s_) of all the photoanodes show small peaks superimposed on the overall declining trends. It is attributed to the activation of catalytic sites.^[^ ^37^ ^]^ These are most likely oxygen vacancies in Al:BVOs.^[^ ^38^ ^]^ This *C*_s_ is related to hole trap states, which are surface defects or energy levels that temporarily capture photogenerated holes before their transfer to the electrolyte, and interact with surface capacitance through surface states (C_ss).^[^ ^39^, ^40^ ^]^ These trap states act as recombination centers, accelerating electron‐hole recombination and reducing photocurrent efficiency. Simultaneously, they contribute to surface state capacitance (C_ss), as trapped charges accumulate at the interface. As Al is incorporated in BiVO4, the *C*_s_ value decreases dramatically. This indicates that the trap state is reduced by doping. However, as the doping amount increases, the *C*_s_ value increases. This increase is due to the formation of excessive oxygen vacancy on the surface.

### 2.4 PEC Performance of Al:BVO

**Figure 4:**
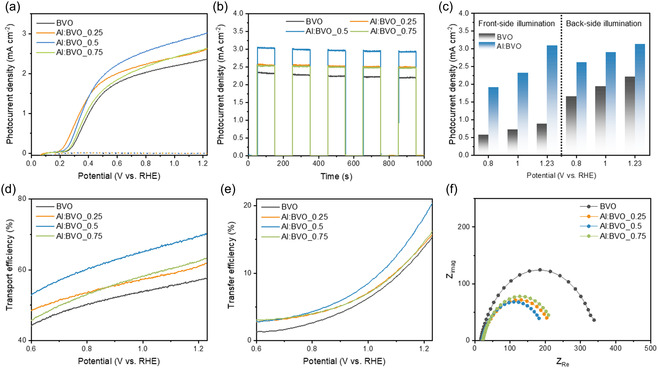
a) LSV curves of BVO and Al:BVOs. b) TPC profile of BVO and Al:BVOs under chopped light illumination. c) Specific photocurrent density of BVO and Al:BVO depending on the illumination direction. d) Charge transport efficiency (*η*_transport_), e) Charge transfer efficiency (*η*_transfer_) of BVO and Al:BVOs. f) Nyquist plot of all the samples at 1.23 *V*_RHE_.

The PEC water oxidation performance of Al:BVOs was evaluated under phosphate buffer electrolyte (pH 7). As shown in **Figure** **4**a, the photocurrent densities at 1.23 *V*_RHE_ were 2.37, 2.62, 3.02, and 2.64 mA cm^−2^, for BVO, Al:BVO_0.25, Al:BVO_0.5, and Al:BVO_0.75, respectively. In spite of low carrier concentration and low conductivity, Al:BVOs exhibited enhanced PEC performance compared to BVO. Especially, Al:BVO_0.5 outperformed the other samples, exhibiting a 38% increase in photocurrent density compared to BVO. This enhanced PEC performance of Al:BVO is ascribed to enlarged SCL and increased charge carrier lifetime. However, the photocurrent density of Al:BVO_0.75 is lower than that of Al:BVO_0.5, which is attributed to Al ions serving as the recombination center and low conductivity. TPC measurements under chopped light illumination were performed at 0.8 *V*_RHE_ (Figure 4b). All photoanodes produced sensitive and repeated photoresponses with light illumination. Photocurrent density subsequently decreased under illumination due to the recombination of EHP until the charge generation and recombination rate reached saturation. Therefore, a slow decay in photocurrent density is related to a lower recombination rate and prolonged carrier lifetime. The photocurrent retention of Al:BVOs during 100 s in Figure S8, Supporting Information, was 97.3, 98.3, 99, and 98.9% for BVO, Al:BVO_0.25, Al:BVO_0.5, and Al:BVO_0.75, respectively, signifying that Al dopants play a positive role in suppressing the recombination of EHP.

The difference between photocurrent density under front‐side irradiation (*J*_front_) and back‐side irradiation (*J*_back_) is investigated to determine the limiting factor for PEC performance.^[^ ^41^ ^]^ Under front‐side illumination, holes are generated near the semiconductor/electrolyte interface, while electrons must travel across the entire film to reach the conducting substrate. In contrast, with back‐side illumination, the situation is reversed; holes now cover the greatest distance to reach the surface. The lowest photocurrent under front‐side illumination indicates that electron transport is the limiting factor in the performance. In Figure 4c and Figure S9, Supporting Information, *J*_front_ of BVO is much lower than *J*_back_, with a value of 0.29 for *J*_front_/*J*_back_ at 1.23 *V*_RHE_, which implies that electron transport is the limiting factor of BVO. In contrast, Al:BVO has a value of 0.71 for *J*_front_/*J*_back_, indicating that excellent electron transport is achieved in Al:BVOs.

To further investigate the charge separation of Al:BVOs in bulk, the transport efficiency (*η*_transport_), which is the fraction of the photo‐generated holes that reach the surface, is analyzed. As shown in Figure 4d, the *η*_transport_ values for Al:BVO_0.25, Al:BVO_0.5, and Al:BVO_0.75, were 61.9, 70.18, and 63.31% at 1.23 *V*_RHE_, respectively, which were higher than that of BVO (57.73%). These results support that lower charge concentration by doping engineering can effectively improve charge transport within the bulk of BVO, enhancing charge separation. The charge transfer efficiency (*η*_transfer_), which is the fraction of photo‐generated holes participating in electrochemical reaction at the surface, was also analyzed by measuring PEC performance without a hole scavenger (Figure S10, Supporting Information). The determined *η*_transfer_ is presented in Figure 4e. Al:BVO_0.5 exhibited the highest value of *η*_transfer_ (20.2% at 1.23 *V*_RHE_) compared to that of the BVO (15.5%). To further study charge transfer at the surface of Al:BVOs, electrochemical impedance spectroscopy (EIS) was employed. The determined charge transfer resistance (*R*_ct_) values from the Nyquist plot at 0.6 *V*_RHE_ (Figure 4f) were 443, 421, 369, and 428 Ω for BVO, Al:BVO_0.25, Al:BVO_0.5, and Al:BVO_0.75, respectively. Based on the above results, it is concluded that introducing Al dopant in BVO not only suppresses the charge recombination in bulk, facilitating charge transport from the bulk into the surface, but also promotes charge injection at the photoanode/electrolyte interface, enhancing the PEC performances.

### 2.5 Charge Carrier Dynamics of BiVO_4_ Depending on Doping Types

**Figure 5:**
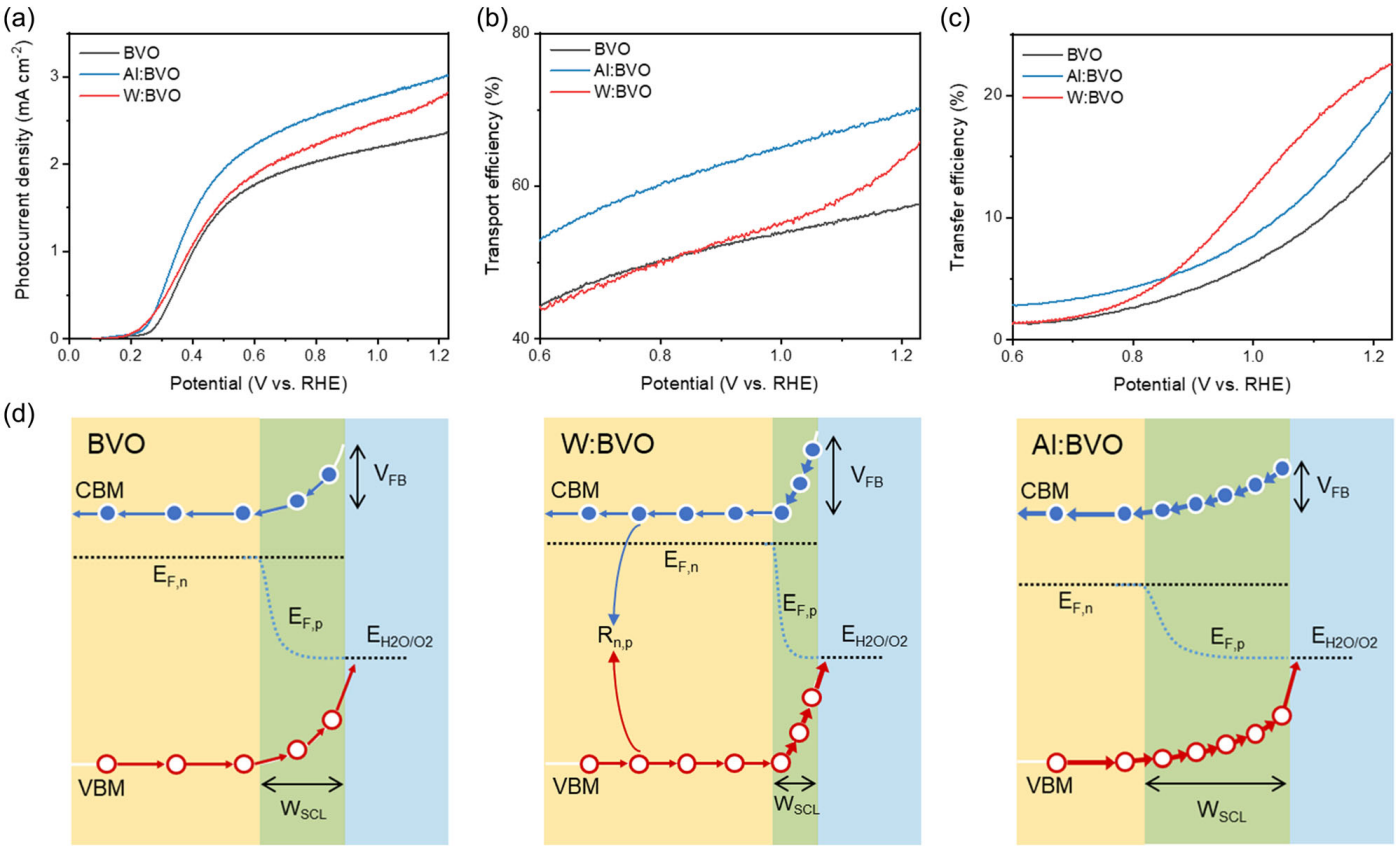
a) LSV curves of BVO, Al:BVO and W:BVO. b) Charge transport efficiency (*η*_transport_), c) charge transfer efficiency (*η*_transport_). d) Schematic illustration of band structure and band bending of BVO, W:BVO, and Al:BVO under illumination with electrolyte.

Conventional donor ion doping enhances n‐type semiconductor properties, while acceptor ion doping reduces carrier concentration, which reduces n‐type properties. These different doping mechanisms affect charge transport and charge transfer. Therefore, to investigate which doping strategy favors charge transport and charge transfer, BVO with acceptor ion (Al^3+^) doping was compared to BVO with conventional donor ion (W^6+^) doping. For comparison, 0.5 at% W‐doped BVO (W:BVO) was fabricated in the same way as BVO. The M–S plot in Figure S11a, Supporting Information, shows that the slope of W:BVO is decreased, indicating an increased carrier concentration in W:BVO compared to BVO. When considering SCL, an increase in carrier concentration of W:BVO led to a reduction in *W*_SCL_, confining it to 3.34 nm at 1.23 *V*_RHE_ (Figure S11b, Supporting Information). The PEC performance of BVO with different doping was evaluated through the linear sweep voltammetry (LSV) curves in **Figure** **5**a. W:BVO exhibited excellent PEC performance compared to BVO with a photocurrent density of 2.83 mA cm^−2^ at 1.23 *V*_RHE_, which confirms that increased carrier concentration and electrical conductivity are effective in improving PEC performance, as previously reported. Meanwhile, Al:BVO exhibited a photocurrent density of 3.02 mA cm^−2^, which is superior to W:BVO. This demonstrates that reducing the carrier concentration by incorporating acceptor ion doping could also be an effective strategy for improving PEC performance. Furthermore, Al:BVO showed enhanced stability compared to W:BVO (Figure S12, Supporting Information). This results from enlarged SCL, increased diffusion length, and extended carrier lifetime due to the reduced carrier concentration. To investigate the charge carrier dynamics depending on doping type, the *η*_transport_ and *η*_transfer_ were analyzed. In Figure 5b, Al:BVO showed a higher *η*_transport_ compared to BVO, while W:BVO did not show a significant change compared to BVO. Even in the lower voltage region, the efficiency of W:BVO is lower than BVO, which is due to the thinner SCL. However, W:BVO showed higher *η*_transfer_ than Al:BVO and BVO (Figure 5c).

Combined with the analysis results, Figure 5d suggests that donor ion doping (W^6+^) in BVO with confined and steeped SCL is effective for charge injection at the interface but has little effect on transport due to significant impurity scattering. Furthermore, with too thin SCL, there is a higher chance that the EHP will recombine before it can contribute to the PEC reaction, lowering efficiency. On the contrary, acceptor ion doping (Al^3+^) in BVO improves the charge separation both at the surface and in bulk simultaneously. This not only enables facile charge transport in bulk but also allows the hole to participate in electrochemical reactions on the surface, which is mainly attributed to the widened SCL and prolonged carrier time. In addition, widened SCL enables effective utilization of incident light. Considering that electron transport is a limiting factor of BVO, the acceptor ion doping strategy is effective for improving charge transport efficiency and PEC performance.

## 3 Conclusion

In summary, A novel strategy to fabricate high‐performance BVO photoanode via acceptor ion doping is demonstrated. The introduction of acceptor ion Al^3+^ in the BVO lattice modulates the carrier concentration of BVO. The reduced carrier concentration improves the charge carrier dynamics, increasing the diffusion length and carrier lifetime. Furthermore, the SCL is widened, enabling a facile charge transport from bulk to surface. This improved charge carrier dynamics of BVO induced by Al doping enhances the PEC performance compared to BVO. Furthermore, the relationship between the doping type and PEC performances is investigated with Al:BVO and W:BVO. Donor ion doping in W:BVO has a negative effect on charge transport but is effective in enhancing charge injection at the interface. In contrast, acceptor ion doping in Al:BVO improves charge transport efficiency both at the surface and within the bulk of the material, which is a key the limiting factor for PEC performance of BVO. The strategy presented here could provide a new avenue for the efficient photoelectrode design.

## 4 Experimental Section

4.1

4.1.1

##### The Fabrication of Al‐Doped BVO Photoanode

BiVO_4_ photoanode was synthesized using the electrodeposition method. 100 mL of 0.03 M Bi(NO_3_)_3_·5H_2_O solution and 0.3 M KI solution was prepared. The pH of this mixed solution was adjusted to 1.7 by adding HNO_3_ (solution 1). The 40 mL of absolute ethanol with 0.23 M *p*‐benzoquinone was prepared and mixed with solution 1 by vigorous stirring. A three‐electrode system was introduced for electrodeposition, composed of an Ag/AgCl (saturated in 3 M KCl) reference electrode, a Pt counter electrode, and a fluorine‐doped tin oxide (FTO) glass as the working electrode. The constant bias of −0.1 V (versus Ag/AgCl) was applied for 3 min. After the electrodeposition, the resulting BiOI‐coated FTO glass was washed with distilled water and ethanol. Subsequently, 0.2 M of vanadyl acetylacetonate was dissolved in dimethyl sulfoxide (DMSO) and dropped onto the as‐prepared BiOI electrode. Then, the coated electrode was annealed at 450 °C for 3 h. Finally, the BiVO_4_ electrode was dipped into 1 M NaOH to remove excess V_2_O_5_ byproduct. In order to fabricate doped BVO, Al(NO_3_)_3_·9H_2_O and (NH_4_)_6_H_2_W_12_O_40_·xH_2_O were added in DMSO containing 0.2 M of vanadyl acetylacetonate with the intended molar ratio.

##### Measurement of PEC Performance

The PEC performance of the BiVO_4_ photoanode was studied using a three‐electrode system controlled by an electrochemical analyzer (model Autolab PGSTAT; Metrohm). A 300 W Xe lamp (6258, Newport) with AM 1.5 and an IR cut filter was used as the light source. Light intensity was calibrated to 1 sun (AM 1.5 G, 100 mW cm^−2^). The PEC performance was characterized under a 0.5 M potassium phosphate buffer solution (KPi, pH = 7) with or without 1 M Na_2_SO_3_ as a hole scavenger. In order to remove dissolved oxygen molecules from the electrolyte, the electrolyte was purged with nitrogen gas for 30 min before measurement. The electrode potential was calibrated against the reversible hydrogen electrode (RHE) by using the equation, *E*(RHE) = *E*(Ag/AgCl) + 0.059 pH + *E* ^o^ (Ag/AgCl), where *E* ^o^ (Ag/AgCl) was 0.1976 V at 25 °C. The photogenerated current density was measured by LSV at a scan rate of 5 mV s^−1^. EIS was measured in the frequency range between 0.1 Hz–100 kHz at an amplitude of 20 mV. Mott–Schottky measurements of Al‐doped BVO (Al:BVO) were performed at a frequency of 1 kHz under 0.5 M KPi solution.

## Conflict of Interest

The authors declare no conflict of interest.

## Author Contributions

**Jiseok Kwon**: conceptualization (lead). **Heechae Choi**: formal analysis (supporting). **Seunggun Choi**: formal analysis (supporting). **Jooheon Sun**: methodology (supporting); supervision (supporting); **Hyuksu Han**: supervision (supporting); visualization (supporting); writing—review and editing (supporting). **Ungyu Paik**: conceptualization (lead); funding acquisition (lead); supervision (lead); writing—review and editing (lead). **Junghyun Choi**: conceptualization (supporting); funding acquisition (lead); supervision (lead); validation (lead); writing—review and editing (lead). **Jiseok Kwon** and **Heechae Choi** contributed equally to this work.

## Supporting information

Supplementary Material

## Data Availability

The data that support the findings of this study are available in the supplementary material of this article.
