# Supplementary material for: Improved Charge Carrier Dynamics by Unconventional Doping Strategy for BiVO4 Photoanode
Source: Small Sci. 2025 May 19;5(7):2500051. doi: 10.1002/smsc.202500051 (PMC12257881; doi:10.1002/smsc.202500051)
Supplement: Supplementary file 1 — Supplementary Material [file SMSC-5-2500051-s001.pdf]

Supplementary Information for

Improved Charge Carrier Dynamics by Unconventional Doping Strategy for $BiVO_4$ Photoanode

Jiseok Kwon,[1,§] Heechae Choi,[2,§] Seunggun Choi,[1] Jooheon Sun,[1] Hyuksu Han,[3] Ungyu Paik,[1,] Junghyun Choi[4,*] and Taeseup Song [1,5,*]*

[1]*Department of Energy Engineering, Hanyang University, 222 Wangsimni-ro, Seongdong-gu, Seoul 04763, Republic of Korea*

[2]*Department of Chemistry, Xi'an Jiaotong-Liverpool University, Suzhou 215123, China*

[3]*Department of Energy Science, Sungkyunkwan University, 2066 Seobu-ro, Jangan-gu, Suwon-si, Gyeonggi-do 16419, Republic of Korea*

[4]*School of Chemical, Biological and Battery Engineering, Gachon University, Seongnam-si, Gyeonggi-do 13120, Republic of Korea*

[5]*Department of Battery Engineering, Hanyang University, 222 Wangsimni-ro, Seongdong-gu, Seoul 04763, Republic of Korea*

[§]*These authors equally contributed to this work.*

*Corresponding authors E-mail: upaik@hanyang.ac.kr, junghchoi@gachon.ac.kr, tssong@hanyang.ac.kr

Experimental details

Materials characterization

The morphology of the catalysts was observed using field emission scanning electron microscopy (FE-SEM, JEOL JSM07600F). X-ray diffraction patterns were recorded by Rigaku's SmartLab instrument. Transmission electron microscopy (TEM, JEOL JEM-2100F) was used and the data were further analyzed by Gatan microscopy suite software version 3 for selected-area electron diffraction (SAED) patterns. The valence state and components of the elements were obtained from X-ray photoelectron spectroscopy (XPS, VG Microtech ESCA2000). Fourier transform infrared spectra of catalysts were measured by Fourier transform infrared spectroscopy (Nicolet iS50) in the range of 400-4000 cm-1. Raman spectra of catalysts were obtained by Micro Raman Spectrophotometer (NRS-3100) in the range of 600-2700 cm^{-1}. Photoluminescence (PL) spectra were obtained by using spectrometer (F-7000, Hitachi). Time-resolved PL (TRPL) was measured using an inverted-type scanning confocal microscope (MicroTime-200, Picoquant) with a 40 × (air) objective. Excitation source was a single-mode pulsed diode laser (LDH-P-C-375, Picoquant) with approximately 30 ps pulse width and approximately 10 μW power. Time-correlated single-photon counting technique was used to count emission photons. Exponential fitting for the obtained PL decays was performed using the Symphotime-64 software (Ver. 2.2). The work function of each sample was estimated using following equation,

$$\emptyset = h\nu - \left(E_{Cut-off} - E_{FE}\right) \qquad (1)$$

where $h\nu = 21.2$ eV (He I source), E_{FE} is fermi level.

Analysis of carrier concentration

Mott-schottky measurement of Al doped BVO (Al:BVO) were performed at frequency of 1 kHz under 0.5 M KPi solution. Carrier density of the catalysts was calculated by using following equation;

$$\frac{1}{C^2} = \frac{2}{\varepsilon\varepsilon_o A^2 e N_d}\left(V - V_{FB} - \frac{k_B T}{e}\right) \quad (2)$$

$$\frac{d(C^{-2})}{dV} = \frac{2}{\varepsilon\varepsilon_o A^2 e N_d} \quad (3)$$

where C is capacitance, ε is the relative permittivity of BVO, ε_0 is the absolute permittivity of vacuum, A is the area of the electrode, N_d is the carrier concentration of the BVO and Al:BVOs, V_{FB} is the flat band potential, k_B is the Boltzmann constant, respectively.

Transient Photocurrent (TPC) Measurement

W_{SCL} and L_D values were obtained by TPC measurements performed by the addition of LED light pulses onto AM 1.5G illumination. The LED diode array's power was measured A pulse width of 1 s was generated, and the additional photocurrent at steady state (ΔJ) was measured in a three-electrode cell configuration. The main procedure to find W_{SCL} and L_D is given below, but more details can be found in another study.

Giving a Lambertian absorption and considering negligible reflection losses (with Transmittance = 1 – Absorptance), one can obtain the distance *D,*

$$\mathrm{D} = -\alpha^{-1}\log(1 - A) = \alpha^{-1}\log(1 - \frac{\Delta J}{J_{pulse}}) \quad (4)$$

Assuming that the charge carrier recombination at the surface is negligible with hole scavanger, ΔJ corresponds to the number of hole carriers reaching the surface for oxidation. The distance D, the sum of the L_D and W_{SCL}, represents the total thickness in which

photogenerated holes can react with the scavenger ions and can be calculated through the equation

$$D = W_{SCL} + L_D = \sqrt{\frac{2\varepsilon\varepsilon_o}{eN_d}}\sqrt{V - V_{FB}} + L_D \quad (5)$$

where ε is the relative dielectric constant, ε_0 is the permittivity of vacuum, e is the electronic charge, N_d is the donor concentration of the BVO and Al:BVOs, and V_{FB} is the flat band potential.

The calculation of transport efficiency ($\eta_{tranport}$) and transfer efficiency ($\eta_{transfer}$)

Photogenerated current density (J_{Ph}) can be expressed as follows;

$$J_{Ph} = J_{max}\, X\, \eta_{abs}\, X\, \eta_{sep}\, X\, \eta_{trans} \quad (6)$$

$$J_{abs} = J_{max}\, X\, \eta_{abs} \quad (7)$$

where J_{max} is the maximum photogenerated current density, η_{abs} is absorption efficiency, η_{sep} is charge separation efficiency in the bulk, η_{trans} is the charge transfer efficiency between the surface of the catalyst and electrolyte, respectively. Assuming that the photoconversion efficiency is 100 %, J_{max} and J_{abs} can be expressed as;

$$J_{max} = q\int_0^{\lambda_i} \emptyset_\lambda \, d\lambda \quad (8)$$

$$J_{abs} = q\int_0^{\lambda_i} A_\lambda \emptyset_\lambda \, d\lambda \quad (9)$$

where Φ is photon flux of the AM 1.5 G solar spectrum, λ_i is the wavelength at absorption edge, A_λ is the absorbance, q is the charge of an electron, respectively. Absorbance (A_λ) of the catalysts were obtained by transmittance (T_λ) and diffuse reflectance (R_λ) using the following

equation ($A_\lambda = 1 - T_\lambda - R_\lambda$). For calculating $\eta_{transport}$ and $\eta_{transfer}$, 1 M of the sodium sulfite (Na_2SO_3) is introduced in electrolyte as a hole scavenger. With a hole scavenger, charge transfer between surface of the catalyst and electrolyte can be assumed 100 %. Therefore, $\eta_{tranport}$ and $\eta_{transfer}$ can be expressed as;

$$\eta_{transport} = J_{Ph}^{Sulfite} / J_{abs} \quad (10)$$

$$\eta_{transfer} = J_{Ph} / J_{Ph}^{Sulfite} \quad (11)$$

PEIS characterization

Photoelectrochemical impedance spectroscopy (PEIS) was conducted at direct current (DC) potentials from +0.5 to +1.1 V_{RHE} at a step of 0.05 V and an alternating current (AC) potential of 10 mV with a frequency range of 10 kHz – 0.01 Hz under simulated sunlight (Xe source, AM 1.5G filter, 100 mW cm^{-2}). A typical equivalent circuit was used to fit, comprising two resistances and a constant phase element (CPE): a series resistance, Rs (system resistance); a charge transfer resistance across the semiconductor-liquid inferface, Rct; and an interfacial CPE, Cs.

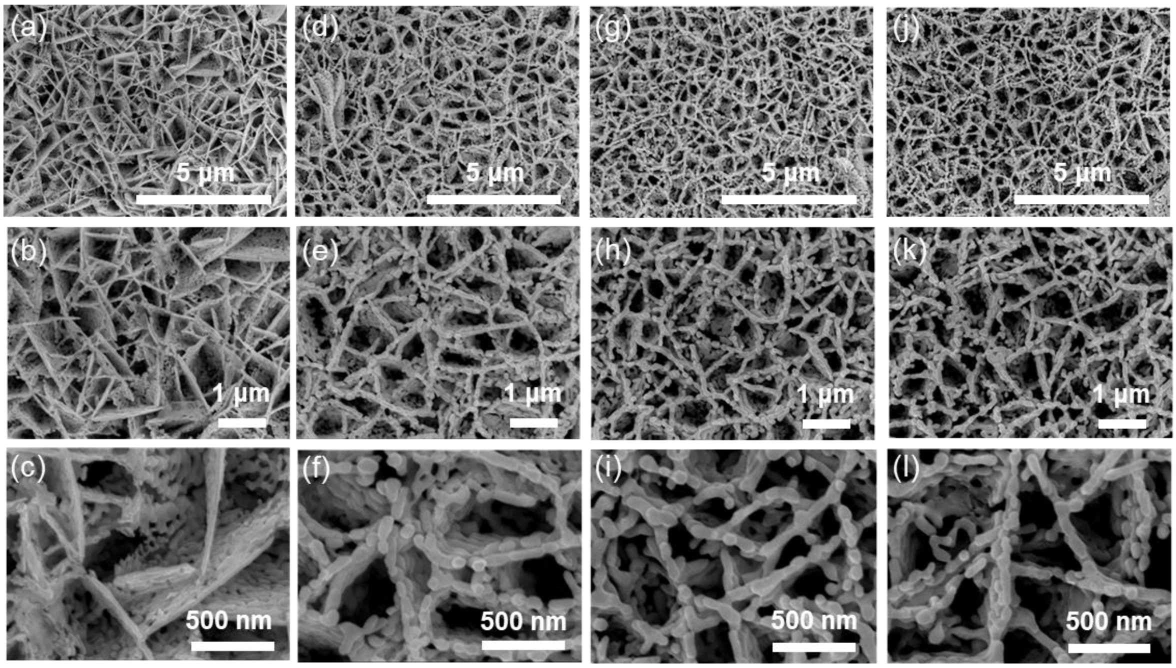

Figure S1. Top view SEM images of (a-c) BVO, (d-f) Al:BVO_0.25, (g-i) Al:BVO_0.5, and (j-l) Al:BVO_0.75.

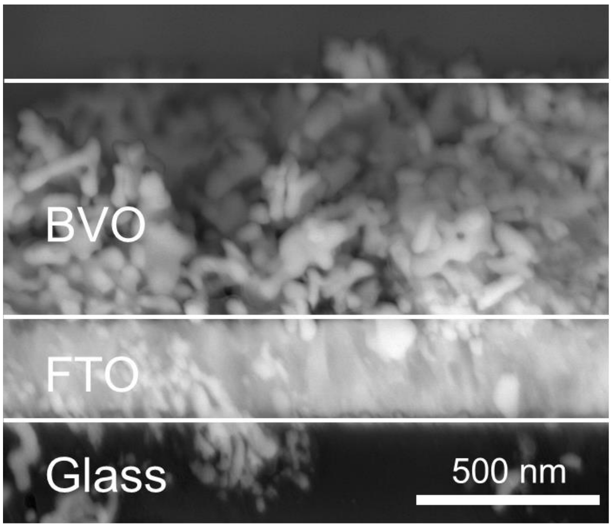

Figure S2. Cross-sectional SEM image of Al:BVO_0.5.

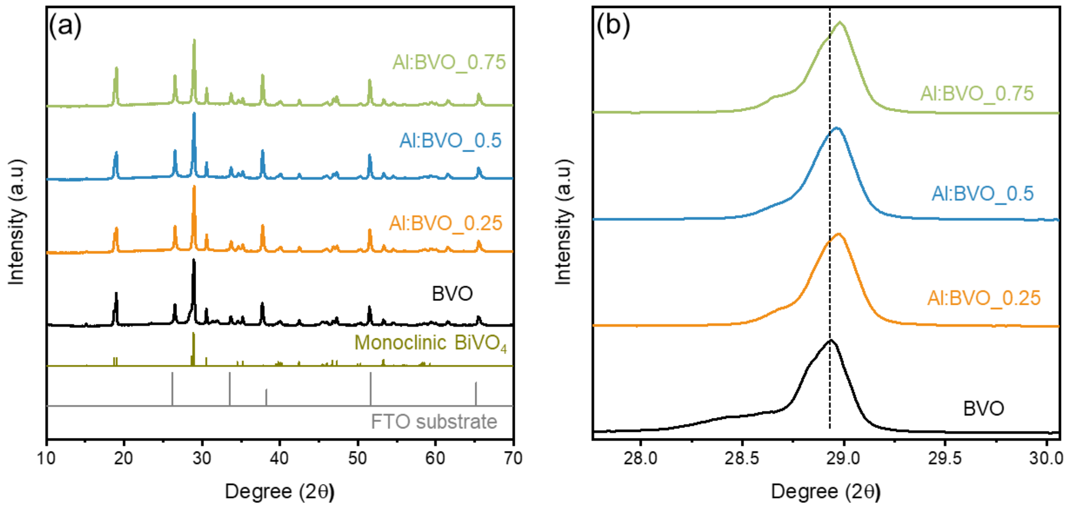

Figure S3. (a) XRD pattern of BVO and Al:BVOs. (b) Magnified XRD view of (112) peak of BVO and Al:BVOs.

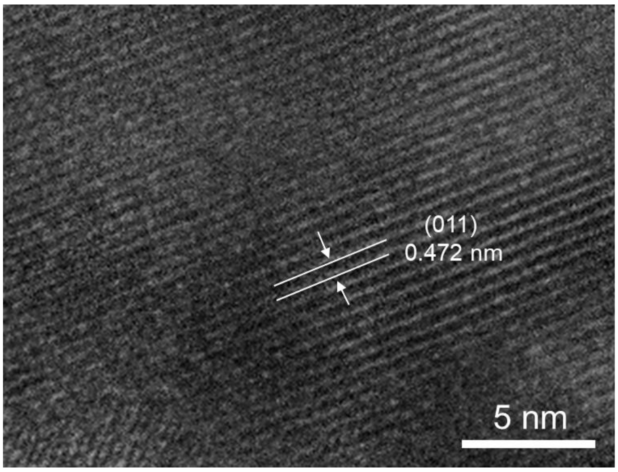

Figure S4. HR-TEM image of BVO

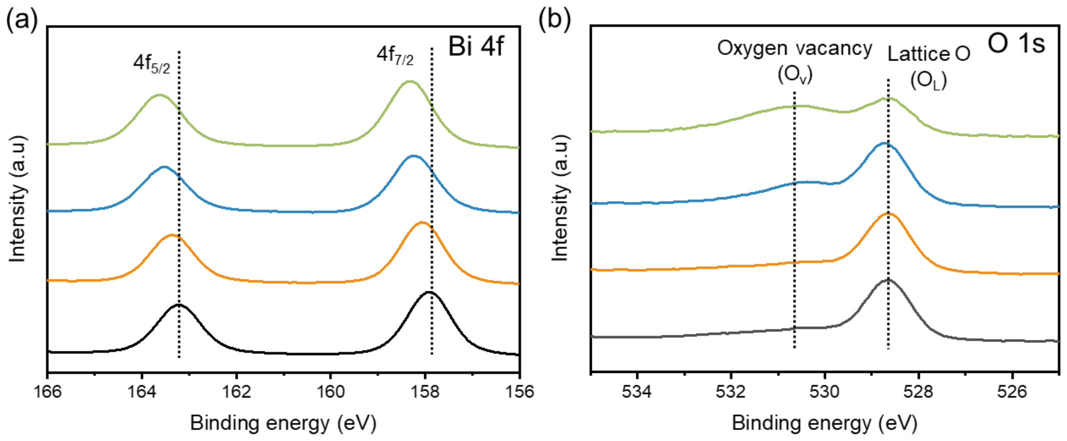

Figure S5. (a) Bi 4f, (b) O 1s spectra of BVO and Al:BVOs.

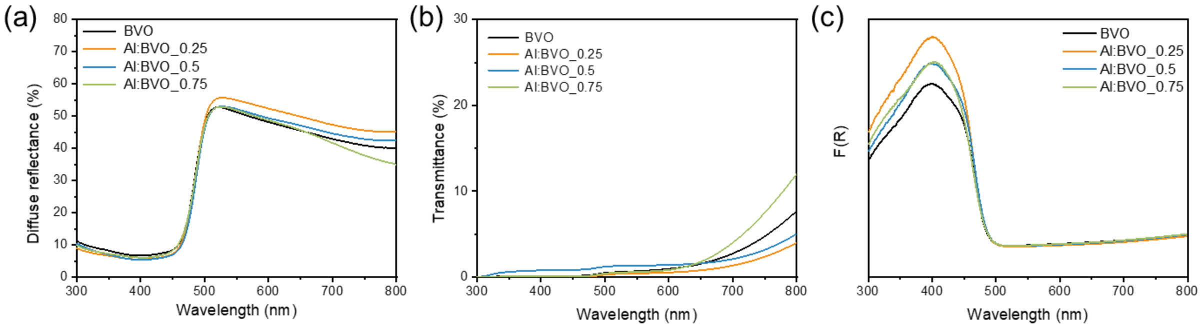

Figure S6. (a) Diffuse reflectance, (b) Transmittance, and (c) Kubelka-Munk function obtained from diffuse reflectance measurements of BVO and Al:BVOs.

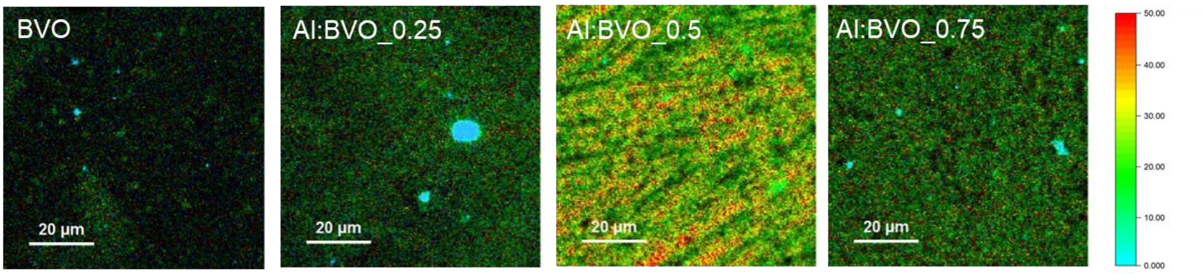

Figure S7. Carrier lifetime image of BVO and Al:BVOs derived from TRPL decay.

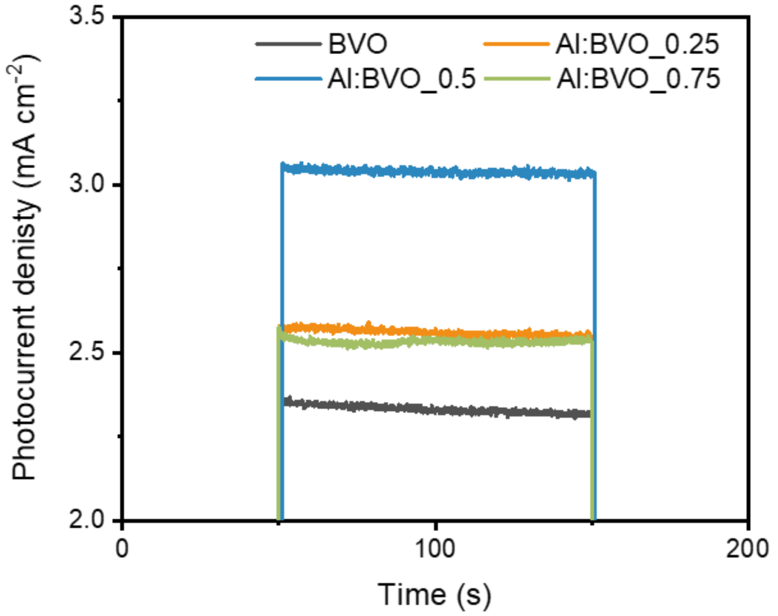

Figure S8. Magnified transient photocurrent profile of BVO and Al:BVOs.

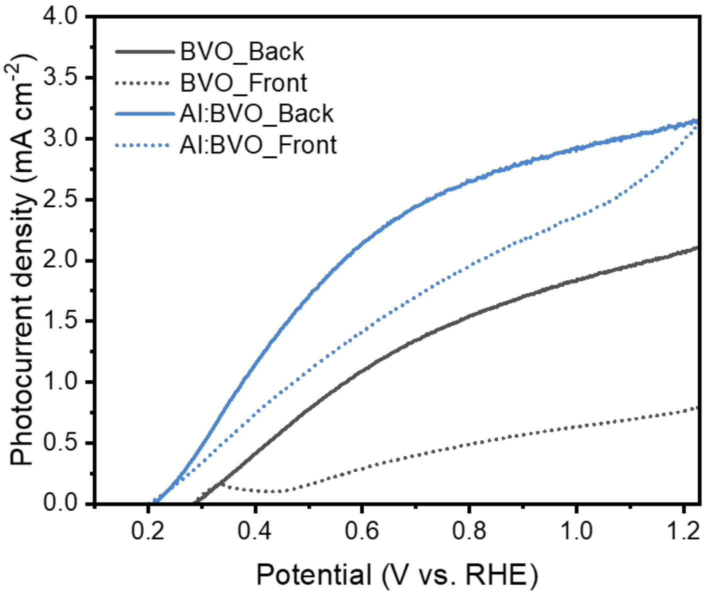

Figure S9. LSV curves of BVO and Al:BVO_0.5 under front-side and back–side illumination.

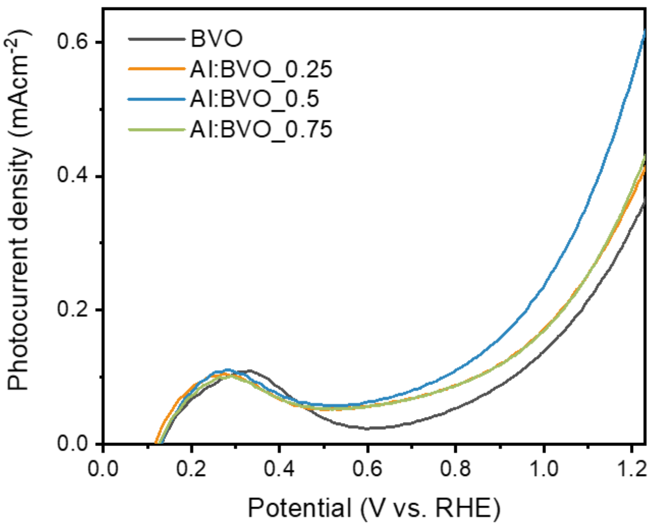

Figure S10. LSV curves of BVO and Al:BVOs without hole scavenger.

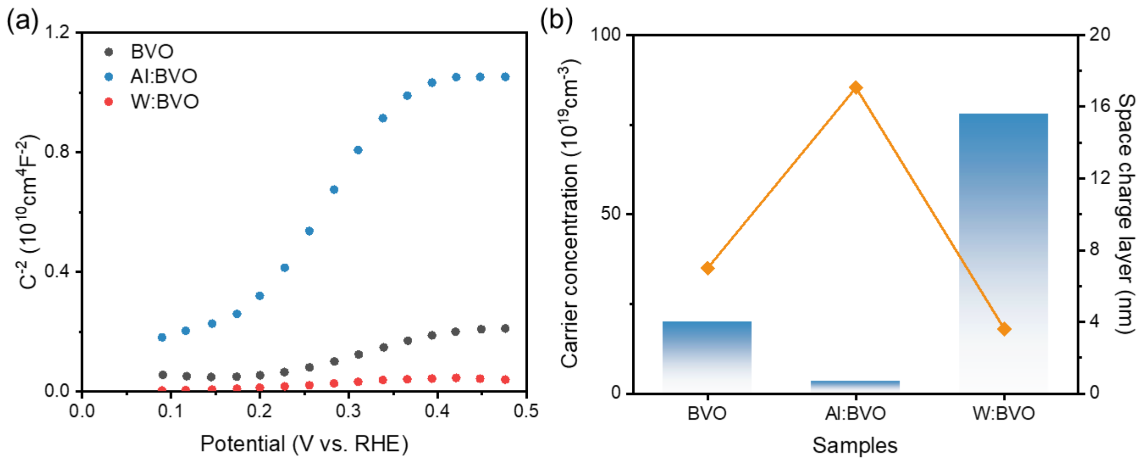

Figure S11. Carrier concentration and width of space charge layer of BVO, Al:BVO and W:BVO.

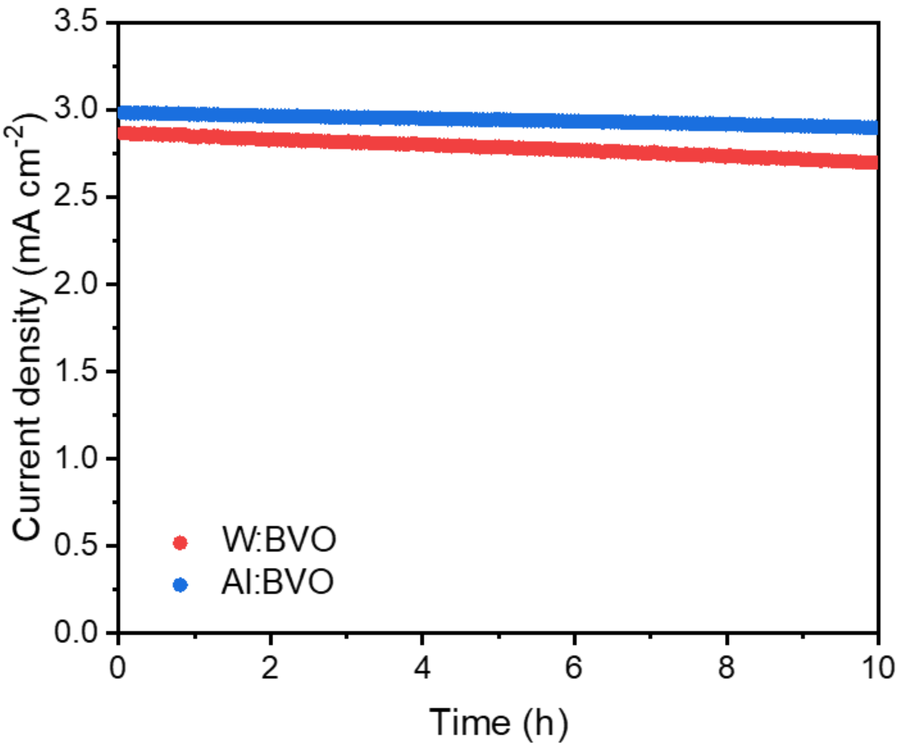

Figure S12. Stability test of W:BVO and Al:BVO.

Table S1. The determined results derived from Mott-Schottky plot

**Sample**	**Flat band potential**	**Nd (Carrier concentration)**	**Space charge layer (nm)**
BVO	0.159	2.05E+20	7.04
Al:BVO_0.25	0.106	5.74E+19	13.43
Al:BVO_0.5	0.101	3.56E+19	17.07
Al:BVO_0.75	0.086	2.57E+19	20.18

Table S2. The determined results derived from UV-DRS and UPS spectra

**Sample**	**Band gap**	**Work function (eV)**	**Ionization energy (eV)**
BVO	2.595	5.5	7.76
Al:BVO_0.25	2.592	5.54	7.81
Al:BVO_0.5	2.586	5.57	7.84
Al:BVO_0.75	2.593	5.62	7.91

Table S3. Parameters of PL decay for Al:BVOs through fitting with four-exponential function.

**Sample**	**A_1**	**τ_1 (ns)**	**A_2**	**τ_2 (ns)**	**A_3**	**τ_3 (ns)**	**A_4**	**τ_4 (ns)**	**τ_{aveg} (ns)**
BVO	0.033	40	5.8	0.242	0.131	9.7	0.6	1.2	14
Al:BVO_0.25	0.052	37	5.9	0.223	0.186	8.7	1.1	0.86	15
Al:BVO_0.5	0.287	56	0.389	2.9	0.581	17	5.1	0.28	37
Al:BVO_0.75	0.111	38	5.9	0.243	0.282	10	0.69	1.1	21

Table S4. Comparison of Al:BVO with other reports.

Sample	Electrolyte	Current density (mA cm^{-2})	References
**Al:BVO**	**0.5 M Posphate buffer**	**3.02 @ 1.23 V**	**This work**
Cs-$BiVO_4$	Borate buffer solution(pH9.5)	3.3	[1]
E-BVO	0.1 M borate buffer (pH ~9)	2.5	[2]
3-2% W:$BiVO_4$	1M PBS	2.2	[3]
ts-BVO	1M Kbi	3.2	[4]
S doped $BiVO_4$	0.5M Na_2SO_4	2.2	[5]
Ni doped $BiVO_4$	1M PBS	2.72	[6]
Mo doped $BiVO_4$	1 M K_2HPO_4 buffer solution (pH 6.8) containing 0.1 M Na_2SO_3	3.5	[7]
Mo doped $BiVO_4$	0.5M Na_2SO_4	2.38	[8]
W doped $BiVO_4$	0.5M Na_2SO_4	0.74	[9]
N doped $BiVO_4$	0.5M Kbi	1.39	[10]

References

1. Tao, C.; Jiang, Y.; Ding, Y.; Jia, B.; Liu, R.; Li, P.; Yang, W.; Xia, L.; Sun, L.; Zhang, B., *JACS Au* **2023,** *3* (7), 1851-1863.

2. Wang, S.; Chen, P.; Yun, J. H.; Hu, Y.; Wang, L., *Angew. Chem. Int. Ed.* **2017,** *56* (29), 8500-8504.

3. Yang, X.; Liang, S.; Miao, J.; Yang, Y.; Zhang, s., *Chemphyschem* **2025,** *26* (1), e202400692.

4. Hwang, S. W.; Jeong, Y. J.; Tan, R.; Saravanan, I.; Han, H. S.; Kim, D. H.; Cho, I. S., *Journal of Advanced Ceramics* **2025**.

5. Peng, Y.; Wu, H.; Yuan, M.; Li, F.-F.; Zou, X.; Ng, Y. H.; Hsu, H.-Y., *Sustainable Energy & Fuels* **2021,** *5* (8), 2284-2293.

6. Chen, M.; Chang, X.; Li, C.; Wang, H.; Jia, L., *Journal of Colloid and Interface Science* **2023,** *640*, 162-169.

7. Chen, L.; Toma, F. M.; Cooper, J. K.; Lyon, A.; Lin, Y.; Sharp, I. D.; Ager, J. W., *ChemSusChem* **2015,** *8* (6), 1066-1071.

8. Parmar, K. P. S.; Kang, H. J.; Bist, A.; Dua, P.; Jang, J. S.; Lee, J. S., *ChemSusChem* **2012,** *5* (10), 1926-1934.

9. Zhao, X.; Hu, J.; Chen, S.; Chen, Z., *Physical Chemistry Chemical Physics* **2018,** *20* (19), 13637-13645.

10. Wang, A.; Chen, Y.; Liu, X.; Li, R.; Zhang, Z.; Zhang, F.; Cao, D.; Gao, Z.; Mi, B., *ACS Applied Materials & Interfaces* **2025**.

Table S4. Comparison of Al:BVO with other reports.

Sample	Electrolyte	Current density (mA cm^{-2})	References
**Al:BVO**	**0.5 M Posphate buffer**	**3.02 @ 1.23 V**	**This work**
Cs-$BiVO_4$	Borate buffer solution(pH9.5)	3.3	[1]
E-BVO	0.1 M borate buffer (pH ~9)	2.5	[2]
3-2% W:$BiVO_4$	1M PBS	2.2	[3]
ts-BVO	1M Kbi	3.2	[4]
S doped $BiVO_4$	0.5M Na_2SO_4	2.2	[5]
Ni doped $BiVO_4$	1M PBS	2.72	[6]
Mo doped $BiVO_4$	1 M K_2HPO_4 buffer solution (pH 6.8) containing 0.1 M Na_2SO_3	3.5	[7]
Mo doped $BiVO_4$	0.5M Na_2SO_4	2.38	[8]
W doped $BiVO_4$	0.5M Na_2SO_4	0.74	[9]
N doped $BiVO_4$	0.5M Kbi	1.39	[10]
